# A review of a 13-month period of FilmArray Meningitis/Encephalitis panel implementation as a first-line diagnosis tool at a university hospital

**DOI:** 10.1371/journal.pone.0223887

**Published:** 2019-10-24

**Authors:** Agathe Boudet, Alix Pantel, Marie-Josée Carles, Hélène Boclé, Sylvie Charachon, Cécilia Enault, Robin Stéphan, Lucile Cadot, Jean-Philippe Lavigne, Hélène Marchandin

**Affiliations:** 1 U1047, INSERM, Montpellier University, Department of Microbiology, Nîmes University Hospital, Nîmes, France; 2 Department of Microbiology, Nîmes University Hospital, Nîmes, France; 3 U1047, INSERM, Montpellier University, Department of Infectious Diseases, Nîmes University Hospital, Nîmes, France; 4 Laboratory of Medical Biology, Alès General Hospital, Alès, France; 5 HydroSciences Montpellier, CNRS, IRD, Montpellier University, Department of Microbiology, Nimes University Hospital, Nîmes, France; University of KwaZulu-Natal, SOUTH AFRICA

## Abstract

Early diagnosis and treatment of meningitis and encephalitis is essential for reducing both their morbidity and mortality. The FilmArray^®^ Meningitis/Encephalitis (FA-M/E) panel is a recently available molecular tool allowing the simultaneous detection of 14 pathogens in about one hour. We evaluated its routine use over a 13-month period at Nîmes University Hospital, France. Cerebrospinal fluid (CSF) specimens were prospectively analyzed, independently of cell count; results were retrospectively analyzed and positive results compared to clinical and microbiological data. Among the 708 patients included (734 CSF samples), 89 (12.6%) had a positive FA-M/E panel, 71 (80%) for a viral pathogen and 18 (20%) for a bacterial pathogen. Enterovirus and HHV-6 were the main detected pathogens. Mean time-to-results was 1h46mn. Four non-clinically relevant results were detected (3 HHV-6 and 1 *Haemophilus influenzae*) on the basis of inconsistent clinical and/or biological data, and/or after visualization of melting curves. No CSF pleocytosis was observed in 11% of the patients with a positive FA-M/E panel. For the 18 patients with a positive FA-M/E panel for a bacterial pathogen, five (28%) had CSF samples showing a positive Gram stain allowing an early diagnosis of bacterial infection and 67% had CSF displaying a positive culture. Altogether the panel detected 5 cases of bacterial M/E (29%) not diagnosed by culture. Despite undeniable advantages, mainly ease of use, quick result availability, and an extremely low rate of invalid results, measures should be implemented to limit false-positive results due to contamination and a careful interpretation based on the overall data for each patient is required.

## Introduction

Meningitis and encephalitis (M/E) are emergency situations of high clinical concern as they are life-threatening and may generate central nervous system sequelae [1,2]. Their management is complicated by the wide range of microorganisms that can be involved. This means a wide range of management strategies ranging from an indispensable early and adequate antibacterial or antiviral therapy to the absence of any antimicrobial prescription [3–5]. Classically, bacterial diagnosis is based on Gram staining and culture while viruses are detected by molecular approaches and *Cryptococcus spp*. by antigen detection and fungal culture [6]. Recently, molecular tools allowing a syndromic approach to M/E diagnosis have become available. As the results are available in around 1 hour, these approaches are of great interest despite their costs, considering the clinical importance of M/E and the potential for improvement in adequate antimicrobial treatment [7].

The first FDA-approved (October 2015) assay was the FilmArray^®^ Meningitis/Encephalitis (FA-M/E) panel (BioFire Diagnostics, a bioMérieux Company, Salt Lake City, UT) allowing the simultaneous detection of 14 pathogens (listed in Table 1) recognized for their ability to cause community-acquired M/E, and M/E in immunocompromised populations. The FA-M/E panel requires 200μl of cerebrospinal fluid (CSF). The test process includes a nucleic acid extraction/purification stage, a first round of highly multiplexed amplification of all targets, followed by a nested-amplification of each target performed on the array in triplicate. The FilmArray system automatically interprets the three results obtained as three independent melting curves to give a unique final result. Despite high-level miniaturized technology, use of the system does not require any specific skills in molecular-based methods [8] requiring only microbiological safety precautions. Handling time and time-to-results reported by the manufacturer are 2 minutes and 1 hour, respectively.

**Table 1:** FilmArray M/E assay results according to detected pathogen, patient medical unit and CSF analysis parameters.

	Positive FA-M/E assay		Patient with positive FA-M/E assay according to medical units							Patient with positive CSF analysis parameters		
	CSF sample (n = 734)	Patient (n = 708)	Emergency unit (n = 350)	Pediatric units (n = 121)	Neonatalogy (n = 31)	ICU (n = 76)	Infectious diseases unit (n = 8)	Neurology (n = 80)	Other units (n = 42)	Positive cytology ^a^	Positive Gram stain	Positive cultures
**Microorganism**												
*Neisseria meningitidis*	5	4 **	1	2	-	1	-	-	-	4/4	2	4
*Streptococcus pneumoniae*	6	4 **	**2**	-	-	**2**	-	-	-	4/4	1	2
*Listeria monocytogenes*	-	-	-	-	-	-	-	-	-	-	-	-
*Escherichia coli* K1	2	2	1	-	1	-	-	-	-	1/1	-	1
*Streptococcus agalactiae*	5	**5**	-	**3**	**2**	-	-	-	-	5/5	2	4
*Haemophilus influenzae*	3	3	**2** ^b^	-	-	1	-	-	-	2/2	-	1
**Total bacteria**	21	18	6	5	3	4	-	-	-	16/16 (100%)	5/18	12/18
Herpes Simplex Virus 1 (HSV-1)	3	2 **	-	-	-	**2**	-	-	-	2/2	na	na
Herpes Simplex Virus 2 (HSV-2)	2	2	2	-	-	-	-	-	-	2/2	na	na
Human Herpes Virus 6 (HHV-6)	8	8	5 ^b^	2 ^b^	-	-	-	**1** ^c^	-	3/7	na	na
Enterovirus (EV)	53	**53**	**24**	**26**	**2**	-	-	-	1 ^d^	47/51	na	na
Cytomegalovirus (CMV)	1	1	-	1	-	-	-	-	-	0/1	na	na
Varicella Zona Virus (VZV)	5	5	1	2	-	-	-	-	**2** ^e^^,^^f^	5/5	na	na
Human Parechovirus (HPeV)	1	1	-	1	-	-	-	-	-	0/1	na	na
**Total viruses**	73 *	72 *	32	32 *	2	2	-	1	3	59/69 (85.5%) *	na	na
*Cryptococcus neoformans*/*gatii*	-	-	-	-	-	-	-	-	-	-	na	-
**Total n (%)**	93 (13) *	89 (13) *	38 (43)	36 (40) *	5 (6)	6 (7)	0	1 (1)	3 (3)	75/85 (83,3) *	5 (27.8)	12 (66.7)

* 94 microorganisms were detected from 93 CSF samples due to one co-detection in a patient hospitalized in the pediatric unit.

** Three patients out of the 27 with repeat tests for clinical reasons had sequential positive results for *Streptococcus pneumoniae* (initial testing and after 11 and 20 days of cefotaxime therapy); *Neisseria meningitidis* (initial testing and on day 13 after 10 and 7 days of cefotaxime and rifampicin therapy, respectively); and Herpes Simplex Virus type 1 (positive on initial testing and on day 12 while on aciclovir therapy).

^a^White blood cells (WBC) ≥5/mm3 in adults, ≥10 mm3 for neonates.

^b^Includes non-clinically relevant results not considered by the physicians in the final diagnosis for corresponding patients as follows: 1 *H*. *influenzae*, 3 HHV-6 (Emergency unit: 2 patients, Pediatric unit: 1 patient).

^c^Neurological symptoms in an immunocompromised adult with HHV-6 DNA detected in blood. CSF: 350 WBC/mm3 (90% of lymphocytes). Initial physician ordering: HSV.

^d^Headaches and fever in a pregnant woman hospitalized in the Gynecology unit. CSF: 245 WBC/mm3 (84% of lymphocytes). Initial physician ordering: *L*. *monocytogenes*.

^e^Monoplegia and aphasia in a patient in the Geriatrics unit. CSF: 90 WBC/mm3 (95% lymphocytes). Initial ordering: EV, HSV, CMV.

^f^Facial palsy and vestibular syndrome in a patient in the Digestive surgery unit CSF: 90 WBC/mm3 (70% lymphocytes). Initial ordering: EV, HSV, CMV, *Borrelia*.

FA-M/E, FilmArray Meningitis/Encephalitis; CSF, cerebrospinal fluid; ICU, intensive care units; N/A, not applicable. Bold numbers indicate the main pathogens in the category considered.

A first large evaluation published in 2016 showed that the panel had performances compatible with early M/E diagnosis [9]. Despite certain pitfalls identified, the authors concluded that the FA-M/E panel was a sensitive and specific test to aid in diagnosis of M/E. However, certain aspects regarding the use of the panel are still a matter of debate as illustrated by a recent point/counterpoint, underlining that prospective studies are still required and that the most appropriate patient population for testing with the FA-M/E panel has yet to be defined [10].

The FA-M/E panel (bioMérieux, Marcy l’Etoile, France) was implemented at our University Hospital in March 2017 and we hereby present a review of results obtained over a 13-month period of routine use.

## Patients & methods

### Ethics statement

This study was conducted at Nîmes University hospital and was approved by the Institutional Review Board under the IRB number 19.01.01.

### Study design

**Fig 1:**
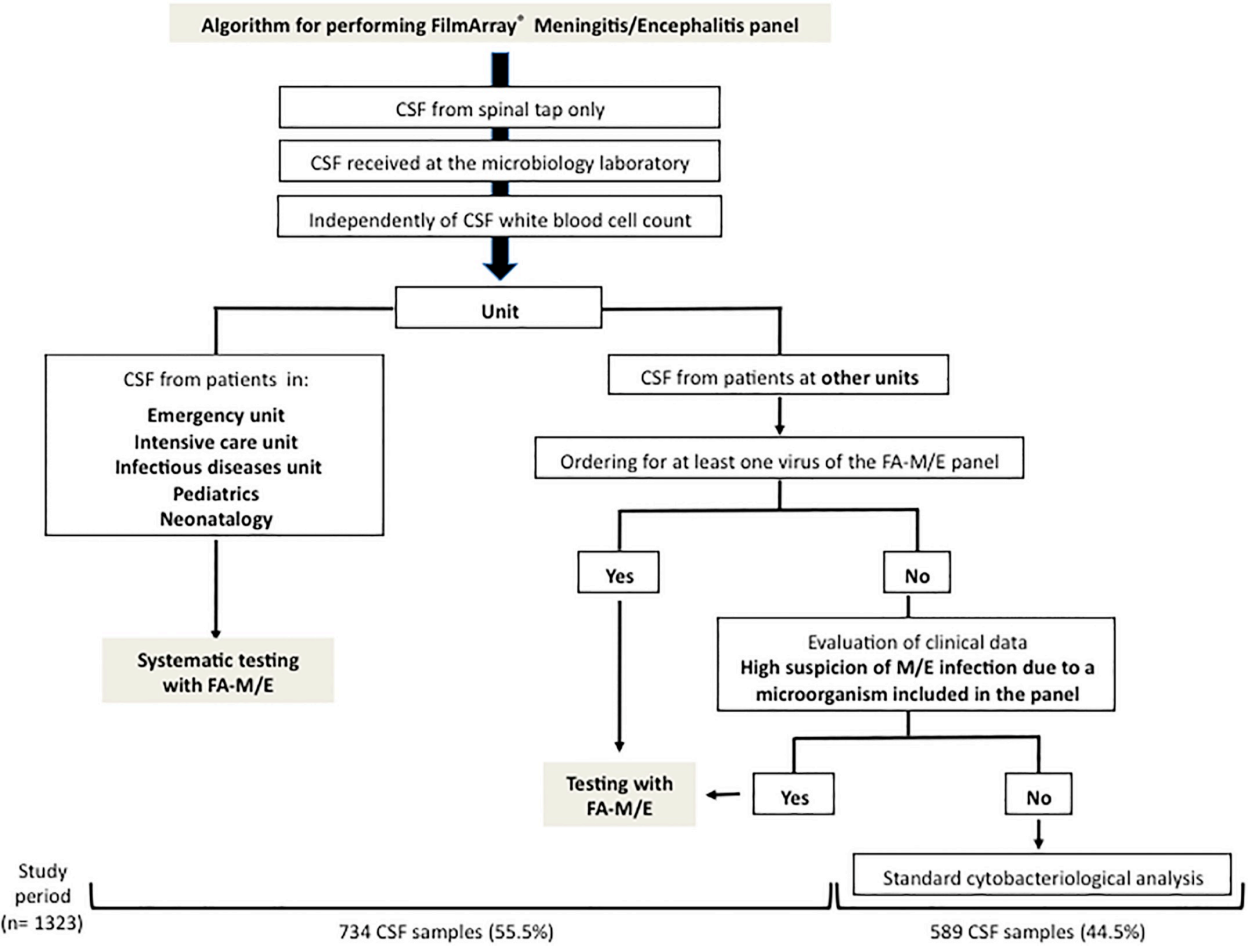
Algorithm for performing the FilmArray Meningitis/Encephalitis assay on CSF samples. All children in the study were put into the “pediatric & neonatology” category.

This study took place at Nîmes University Hospital, a large regional University Hospital (about 2000 hospitalization beds and 130,000 consultations in 2017) without a transplant center, in the South of France. We retrospectively analyzed laboratory data for patients with CSF specimens sampled by lumbar puncture and tested by the FA-M/E panel per physician or microbiologist order between April 3rd 2017 and April 30th 2018. Clinical and complementary laboratory data were retrieved for patients with a positive FA-M/E panel result. The decision for performing FA-M/E panel was independent of the CSF white blood cell (WBC) count and was based on the algorithm presented in Fig 1.

The assay was performed 24 hours/day, 7 days/week. All patients hospitalized in local institutions (general hospital, private clinics) and for whom a CSF sample was sent to our lab during the same period with a prescription for FA-M/E testing were also included.

WBC count and bacteriological cultures were performed according to national/European guidelines [6]. *Cryptococcus* antigen detection and fungal cultures were performed upon request from the clinician according to national/European guidelines [6]. No specific viral genome amplification was routinely performed in addition to the FA-M/E panel at the time of the study as the FA-M/E panel had replaced previous molecular tests.

For CSF samples received from local institutions (general hospital, private clinics) with a specific prescription for the FA-M/E panel, no other analyses were performed at our institution and the results were analyzed separately.

### Pathogen detection result analysis

Positive results were related to clinical data reviewed from medical charts, cytological, bacteriological, and fungal results. Pathogen detection was interpreted in the light of clinical data, cytological, bacteriological, and fungal results in order to identify false-positive results. False-negative results were evaluated for bacterial and fungal pathogens only as no comparative assay for viruses was routinely performed in the laboratory after FA-M/E implementation. In the event of discordant results and CSF availability, confirmatory tests were performed. We also evaluated the chronology of pathogen detection to identify epidemic situations.

### Time-to-results

Time-to-results was defined as the time from receipt of the CSF by the laboratory to the time of reporting the positive or negative result to the clinician. The time was evaluated with laboratory software (Glims^®^, MIPS, Ghent, Belgium).

### FA-M/E panel detailed result review

Melting curves were retrospectively visualized for all CSF analyses with the aim of evaluating the need to examine the curves in the post-analytical stage.

## Results

### Population and sample description

In total, 1323 CSF specimens were received, of which 734 (55%) from 708 patients were subjected to FA-M/E analysis (Fig 1). Patients were 46.6% female (n = 330) and 53.4% male (n = 378) with ages ranging from 1 day to 98 years (mean age: 44 years). Adult patients (n = 556, 78.5%) were majority (mean age: 52.9 years [18–98]) as compared to children (n = 152, 21.5%) (mean age: 3.3 years [1 day-17 years]).

In 27 patients, tests were repeated (two or three analyses) for clinical reasons, with an interval range of 0–47 days. CSF specimens were mostly sampled in patients from the emergency unit (47%) the pediatric/neonatal units (21%), ICUs (12%) and the infectious diseases unit (2%) (Table 1). In total, 82% of the tests were performed on CSF samples sent by units for which systematic testing had been decided whereas 18% were performed for other units, mainly the neurology unit (80 out of 122 analyses, 65.5%).

Blood cell counts could be evaluated for 97.5% (716/734) of CSF samples; 234 samples (32.7%) showed pleocytosis (WBC count ≥ 10/mm3 for neonates, ≥ 5/mm3 for other patients) whereas 59 samples (8.2%) had red blood cell counts of over 1000/mm3.

### Overall FilmArray M/E results and time-to-results

Three samples that did not contain red blood cells yielded invalid results; repeated assay yielded negative results.

A total of 93 CSF specimens (12.7%) sampled in 89 patients (12.6%) were positive using the FA-M/E panel. Instrument running time was 76 minutes. At our institution, mean time-to-results for the clinician was 106 minutes after receipt of the CSF sample at the laboratory. Out of the 27 patients with repeat tests for clinical reasons, three had sequential positive results for *Streptococcus pneumoniae*, *Neisseria meningitidis* or Herpes Simplex Virus type 1 after 12 to 20 days of antimicrobial therapy and in the absence of improvement or worsening of clinical signs (Table 1). The relative significance of detected microorganisms is presented in Table 1.

All further results were presented according to patient number. Higher positive rates were observed in children (27%) than in adult patients (8.6%). For adults, a 10.8% and 7.9% diagnostic yield were observed for patients attending the emergency unit and hospitalized in ICUs, respectively. A higher diagnostic yield was observed for patients in the emergency, infectious diseases, pediatrics and neonatology departments, and ICUs where systematic testing of CSF by FA-M/E had been implemented (14.5%, 85/586) versus patients from other units (3%, 4/122).

Viruses were detected in 71 patients (80% of the patients with positive FA-M/E assay) and bacteria were detected in the remaining 18 patients (20%). Enterovirus (EV) and *Streptococcus agalactiae* were the most prevalent virus and bacterium detected, respectively (Table 1). A single co-detection (EV and HHV-6) was observed for a hemorrhagic CSF specimen sampled in a one-year-old child.

**Fig 2:**
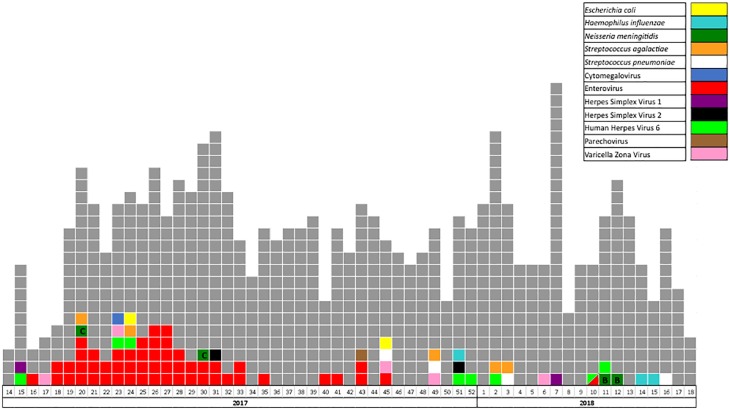
Chronogram presenting the weekly results of the FilmArray Meningitis/Encephalitis panel during the study period. Grey: no pathogen detected. Each colored square represents a patient with positive detection (for the few patients with successive positive detections, only the first positive detection has been considered in the figure). The pathogens detected are indicated by a specific color. A bicolor square indicates the unique co-detection of two pathogens during the study. *N*. *meningitidis* serogroup (B or C) is indicated in the corresponding area.

A chronogram of detections is presented in Fig 2. More than one EV infection was diagnosed per week between early May and mid-August and in October with no other clustered cases, as the two cases of *N*. *meningitis* serogroup B were detected in two unrelated patients.

### Concordance of positive FA-M/E results with routine cytomicrobiological tests, alternative molecular methods, clinical data and prescriptions

Among the 85 patients with positive FA-M/E assay for whom the CSF cytology could be established, 9 (11%) (age range: 4 days—64 years) had CSF with negative WBC count; the microorganism detected was a virus in all cases (Table 1).

For the 18 patients with a bacterial pathogen detected, 28% had CSF samples showing a positive Gram stain allowing early confirmation of the clinical diagnosis and 67% had CSF displaying a positive culture for the species detected by the FA-M/E panel (Table 1). CSF cultures remained negative for 6 of these patients (Table 2). For two of the 6 patients, the CSF had been sampled after treatment initiation. Five patients received antimicrobial regimens against the identified pathogen while one *Haemophilus influenzae* was considered as a contaminant in an adult patient presenting with convulsions and delirium tremens during alcohol withdrawal. Among the 734 CSF samples analyzed, cultures revealed two additional positive samples for bacteria not included in the panel in patients with a high risk for a non-panel target, *i*.*e*., a *Staphylococcus aureus* and a *Streptococcus salivarius* in a healthcare-associated infection and a carcinomatous (esophageal cancer) meningitis, respectively.

**Table 2:** Characteristics of the 6 cases with positive FilmArray Meningitis / Encephalitis assay (FA-M/E) and negative cultures of the cerebrospinal fluid (CSF).

Patient age, unit	Micro-organism detected by FA-M/E	CSF WBC (/mm3) (% of PMN)	CSF RBC (/mm3)	CSF protein level (g/L)	Glycorrha-chia (mmol/L) (ratio ^a^)	Antibiotics before lumbar puncture	CRP (mg/L) (PCT, ng/ml)	Treatment	Blood cultures
52y, ICU	*S*. *pneumoniae*	390 (80%)	660	1.52	2.7	NA	133	Cefotaxime	*S*. *pneumoniae* ^b^
48y, ICU	*S*. *pneumoniae*	656 (90%)	130	5.95	3.4	Yes	73.1 (2.35)	Cefotaxime	*S*. *pneumoniae*
8d, Neonatalogy	*S*. *agalactiae*	8900 (70%)	80	1.89	3.3 (↘)	NA	86.4 (14.5)	Cefotaxime	Negative
21d, Neonatalogy	*E*. *coli* K1	NA	NA	NA	NA	NA	54 (0.1)	Cefotaxime, gentamicine	Negative
70y, ICU	*H*. *influenzae*	30 (10%)	440	0.35	4.4	Yes	108.7	Cefotaxime	NA
36y, Emergency unit	*H*. *influenzae*	8	400	0.7	4.2 (↘)	NA	101	Antibiotic stop	Negative

WBC, white blood cell; PMN, polymorphonuclear neutrophils; RBC, red blood cell; CRP, C-reactive protein; PCT, procalcitonin; NA, not available, either not performed, not determined or not specified.

^a^ ↘, decreased ratio glycorrhachia / glycaemia.

^b^ The patient had a negative *S*. *pneumoniae* antigenuria.

All EV (n = 53 patients), HSV-1 (n = 2), HSV-2 (n = 2), varicella zoster virus (VZV) (n = 5), human parechovirus (HPeV) (n = 1), and cytomegalovirus (CMV) (n = 1) detections were consistent with clinical data. Five of the 8 patients who were positive for Human Herpes Virus-6 (HHV-6) were considered by the physicians to have a viral infection; their age ranged from 1 to 67 years and three of them had CSF displaying pleocytosis (54–350 leukocytes with 70–95% lymphocytes). CSF was available for 6 of these patients and samples were subjected to an artus^®^ HHV-6 RG PCR kit (Qiagen) that confirmed 3 of the detections. It should be noted that the case of co-detection of EV and HHV-6 was confirmed by alternative methods but HHV-6 detection was not considered by the physicians as clinically relevant.

Regarding *Cryptococcus*, 33 samples had a specific prescription of fungal analysis, all of them had negative FA-M/E result concordant with negative antigen detection and fungal culture.

Finally, we evaluated our algorithm of use of the FA-M/E panel. After exclusion of the 4 results that were considered as non-clinically relevant (3 HHV-6, 1 *H*. *influenzae)*, M/E diagnosis was obtained for 9 patients with a negative CSF cytology (10.6% of the patients with available results). For the four patients hospitalized in units not eligible to the systematic FA-M/E testing (Table 1), all had clinical symptoms and biological results that were consistent with the detected pathogen (1 HHV-6, 1 EV and 2 VZV). Importantly, we noted that, in all cases, the initial prescription did not include the etiological agent identified that would have been overlooked if the analyses had been limited to those prescribed by the physicians (Table 1).

### Review of melting curves

**Fig 3:**
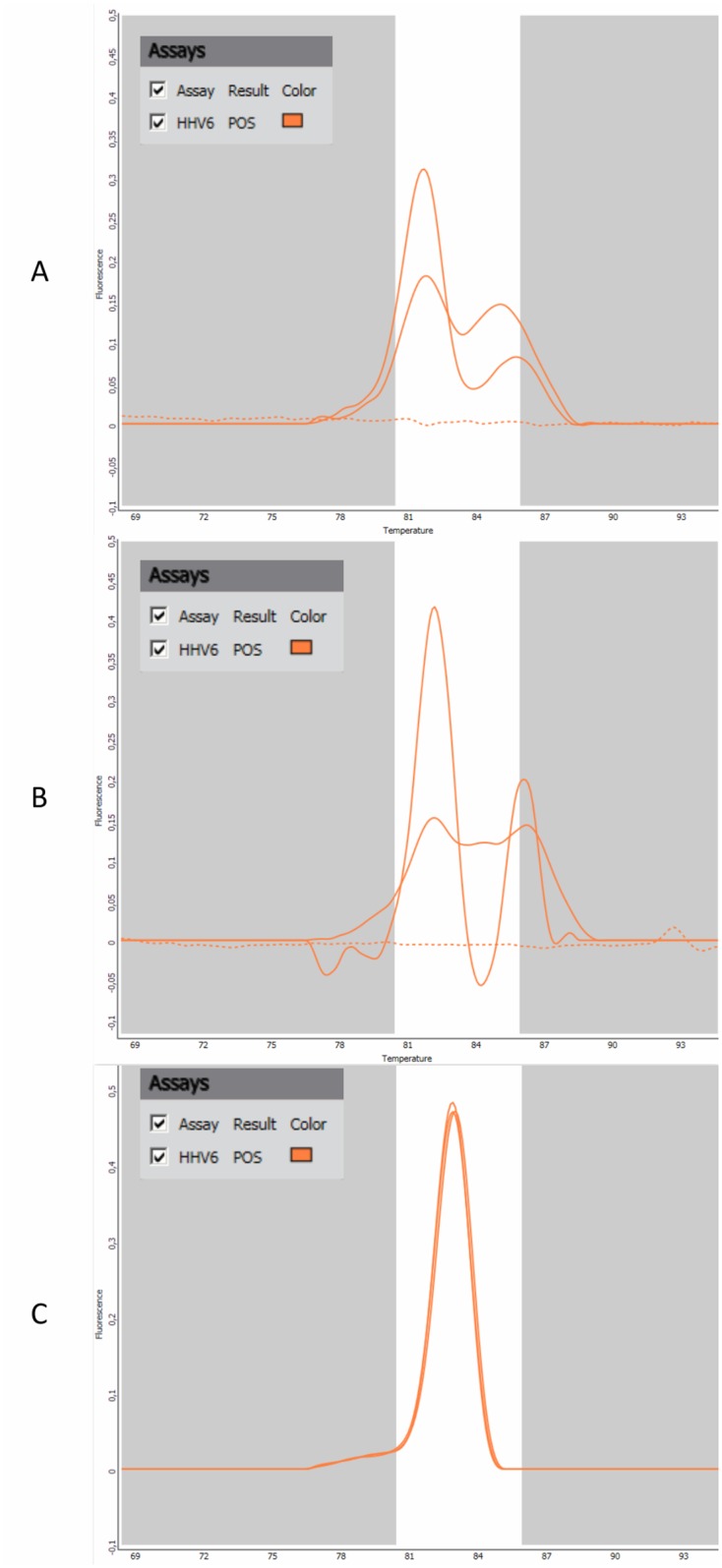
Melting curves for HHV-6 detection declared positive by the system. **A & B.** Noisy melting curves observed for two analyses of CSF with non-confirmed detection of HHV-6 by an alternative method. The two analyses showed that 2 replicates out of the 3 performed by the system were positive. **C.** Melting curves observed after analysis of a CSF sample with confirmed detection of HHV-6 by an alternative method. The 3 replicates performed by the system are positive.

Of all the melting curves retrospectively visualized, atypical melting curves were observed for 2 HHV-6 detections (Fig 3A and 3B) compared to other positive detections (Fig 3C). For both samples, the result was not confirmed by the artus^®^ HHV-6 RG PCR kit. In the first case, a 40-year old man consulting at the emergency unit for suspicion of meningitis, the physician ordered the detection of CMV, EV, HSV and Epstein Barr Virus. The CSF showed a negative cytology, a second assay performed on a CSF sampled the next day was negative, and HHV-6 was not retained in the final diagnosis. For the second patient, a 20-year-old woman consulting at the emergency unit for acute headaches, the CSF showed 62 WBC/mm3 with 70% of lymphocytes and elevated CSF protein level, the HHV-6 detection was considered by the physician and an antiviral treatment administered to the patient.

### Analyses performed for other institutions

A total of 140 specimens from 140 patients (adults, 80%; children, 20%; mean age, 46.6 years) were received from local institutions. A pathogen was detected in 24 (17%) patients: EV (10); *S*. *pneumoniae* (3); *N*. *meningitidis*, *H*. *influenzae*, and VZV (2 each); and *L*. *monocytogenes*, *E*. *coli*, HSV-1, HSV-2, and HHV-6 (1 each). One center notified that *S*. *pneumoniae* and the *E*. *coli* amplification had been considered as false-positive results (WBC count < 5/mm3, negative culture and lack of clinical congruence).

## Discussion

Despite the small number of studies on FA-M/E use currently available (42 studies retrieved with the Mesh terms “FilmArray” and “meningitis” on 17 July, 2019), their comparison is limited by highly diverse study designs, e.g., CSF selection criteria [11,12]; focus on CSF specimens positive or negative by other methods [13,14], on specific population defined by age or clinical condition [15–20], on specific pathogens [17,21–24]; result comparison with other approaches for all or specific discordant results [9,18] or no comparison (most studies). Most of these studies were retrospective, and our literature review only identified four large prospective studies [8,11,25,26]. We present the fifth study on prospective CSF testing with the FA-M/E panel and show an overall positive rate of 12% after exclusion of non-clinically relevant results, consistent with the aforementioned studies. Viruses were the main pathogens identified, in agreement with previous studies showing that 57% [8], 61% [27] or, more frequently, 75–80% of detected pathogens were viruses [9,11,25,26]. The main pathogens detected were EV and HHV-6 as in most of these previous studies. In our study, the dynamics of EV detection was similar to that observed in our country in 2017 (https://cnr.chu-clermontferrand.fr/CNR/Pages/ActivitéCNR.aspx [accessed July 17th, 2019]). Co-detections were previously reported in 0.1–1.45% of the samples and in 1–6% of positive samples [9,11,13,16,19,25,27, this study]. However, although true mixed M/E have been previously described [28], most co-detections were not confirmed by alternative methods applied [9,13,19,29]. In our study, the co-detection of EV and HHV-6 was confirmed by other tests but the HHV-6 detection was considered as non-clinically relevant.

Results were available to the physicians with a median time of less than 2 hours, consistent with previous studies estimating that the median time-to-diagnosis, from collection to result, was about 3 hours [16,19]. This faster time-to-diagnosis compared with conventional methods allows early therapeutic adjustment and, from our experience, also makes the assay efficient for patients hospitalized in other establishments with daily shuttles to our institution. Importantly, as not reported before, we also noticed a very low rate of invalid results (0.5%) and no amplification inhibition for blood-tinged CSF specimens.

Five bacterial M/Es were diagnosed using the FA-M/E panel only, corresponding to 6% of the overall diagnoses and 29% of bacterial meningitis diagnoses. Such an increase in positive results using the FA-M/E panel has been previously described, particularly in the study by Leber *et al*. showing an additional diagnostic yield of 15.5% [9], and in studies re-examining CSF with negative results by other methods showing that 20–23% of patients had a positive FA-M/E assay [14,24]. A potential higher sensitivity of the method, based on nested amplification [24] together with the detection of pathogens after antimicrobial therapy initiation [14, this study], may account for this increased diagnostic yield.

Another advantage of the syndromic approach is the detection of pathogens that would have been missed by routine testing based on the absence of clinical suspicion as observed for four patients in our study. This was also clearly illustrated in the study by Wootton *et al*. who described routine evaluation that did not include VZV or HSV (one patient each) but was detected by the FA-M/E panel [14].

No systematic assessment was performed to determine the trueness of results. False-negative results, in particular, were not evaluated herein as we did not have comparator testing for the viral targets and did not retrieved clinical data for all patients included in the study but only for those with a positive FA M/E panel result. However, false-negative results have been previously documented for several pathogens on the panel (*S*. *agalactiae*, *E*. *coli*, HHV-6, EV, HSV-1, HSV-2, VZV, and *Cryptococcus*) [9,12,13,18,19,30–32]. A viral load close to the limit of detection has been shown to account for some of these false-negative results [32]. Nevertheless, for CSF samples subjected to other detection methods, we did not identify any false-negative results for *Cryptococcus* (33 samples) or bacterial pathogens (all samples), as the two samples that displayed positive cultures and negative FA-M/E assay grew bacteria not included in the panel. On the other hand, relating positive FA-M/E results to clinical data led to the identification of 4 non-clinically relevant positive FA-M/E results (1 *H*. *influenzae* and 3 HHV-6). Not only HHV-6 but also CMV detections must be cautiously interpreted as both viruses are highly prevalent in the population and their amplification might correspond to primary infection, secondary reactivation or to a latent virus [23,33,34], as indicated in one of the system’s warning messages. Despite not having been observed in our study, reactivation of herpes viruses has also been described during bacterial meningitis [35,36]. The importance of correlating FA-M/E results with clinical and other biological data is confirmed in our study. False-positive results have been previously described, accounting for 4% [13], 5.5% [16], 15.6% [9], and 25% [25] of positive results. Regarding potential contamination, clinical and laboratory measures to mitigate false-positive results have been proposed [37]. CSF specimen collection and handling should avoid any contamination by oropharyngeal microbiota whose amplification may lead to false-positive results that may in turn delay the true diagnosis [37]. This warrants clinician information, strict laboratory CSF handling and also the addition of interpretative comments when giving the result to the physician. Based on our systematic review of melting curves, we proposed that: i) it could represent another item to be included in the interpretation stage in the event of FA-M/E panel results not being consistent with clinical and other biological results, ii) visualization of atypical curves in these cases should prompt repeat or alternative testing [30]. However, the interest of checking melting curves must be more thoroughly investigated by further studies.

As the most appropriate patient population for testing with the FA-M/E panel is a point that still requires consideration [10,38], we also discussed the strategy for use of the panel in our laboratory. We found that diagnostic yield in patients hospitalized in units other than those selected for systematic FA-M/E testing were rare, suggesting that use of the panel should be restricted to patients with a high suspicion of M/E in these units to limit unnecessary tests.

Regarding CSF WBC count, 8.2% of patients with M/E diagnosis had acellular CSF. Even higher rates of patients with positive FA-M/E and acellular CSF were reported in the literature: 15% of neonates with bacterial M/E [11], 45.5% of the pediatric population with viral M/E [25], almost 40% of the EV-positive infants, and over 90% of the HPeV-positive infants [16]. Although pleocytosis can be predictive of bacterial meningitis in untreated immunocompetent patients outside the neonatal period, it dramatically lacks sensitivity to predict M/E in other situations. Finally, the fact that selection based on CSF cell counts should not be recommended for FA-M/E testing has recently been reinforced by a modelled economic analysis showing that syndromic testing for all children with suspected M/E is not more expensive as a standard of care and, importantly, that testing only cases with abnormal CSF is not cost-effective [38–41].

## Conclusions

Despite certain limitations to bear in mind when interpreting results (as for all biological diagnosis methods), routine use of the FA-M/E panel presents undeniable advantages, mainly fast results availability for a large panel of pathogens with a small sample volume requirement and simplicity of use. In the context of scarce availability of prospective studies on the FA-M/E panel and the ongoing debate about the most appropriate patient population for testing with the panel, our study provides valuable data on the proposed algorithm for FA-M/E testing. These algorithms appear mandatory, as routine availability of the FA-M/E panel has recently been shown to lead to overutilization of test ordering in patients with little or no suspicion of M/E [26]. In addition, for the first time, we also report an extremely low rate of invalid results and no amplification inhibition for blood-tinged CSF specimens.
